# Weeds, as ancillary hosts, pose disproportionate risk for virulent pathogen transfer to crops

**DOI:** 10.1186/s12862-016-0680-6

**Published:** 2016-05-12

**Authors:** Celeste C. Linde, Leon M. Smith, Rod Peakall

**Affiliations:** Evolution, Ecology and Genetics, Research School of Biology, The Australian National University, Canberra, ACT 2601 Australia

**Keywords:** Population size, Genetic diversity, Migration, Pathogen evolution, Weedy host, Barley, *Rhynchosporium commune*, Fungi

## Abstract

**Background:**

The outcome of the arms race between hosts and pathogens depends heavily on the interactions between their genetic diversity, population size and transmission ability. Theory predicts that genetically diverse hosts will select for higher virulence and more diverse pathogens than hosts with low genetic diversity. Cultivated hosts typically have lower genetic diversity and thus small effective population sizes, but can potentially harbour large pathogen population sizes. On the other hand, hosts, such as weeds, which are genetically more diverse and thus have larger effective population sizes, usually harbour smaller pathogen population sizes. Large pathogen population sizes may lead to more opportunities for mutation and hence more diverse pathogens. Here we test the predictions that pathogen neutral genetic diversity will increase with large pathogen population sizes and host diversity, whereas diversity under selection will increase with host diversity. We assessed and compared the diversity of a fungal pathogen, *Rhynchosporium commune,* on weedy barley grass (which have a large effective population size) and cultivated barley (low genetic diversity) using microsatellites, effector locus *nip1* diversity and pathogen aggressiveness in order to assess the importance of weeds in the evolution of the neutral and selected diversity of pathogens.

**Results:**

The findings indicated that the large barley acreage and low host diversity maintains higher pathogen neutral genetic diversity and lower linkage disequilibrium, while the weed maintains more pathotypes and higher virulence diversity at *nip1*. Strong evidence for more pathogen migration from barley grass to barley suggests transmission of virulence from barley grass to barley is common.

**Conclusions:**

Pathogen census population size is a better predictor for neutral genetic diversity than host diversity. Despite maintaining a smaller pathogen census population size, barley grass acts as an important ancillary host to *R. commune*, harbouring highly virulent pathogen types capable of transmission to barley. Management of disease on crops must therefore include management of weedy ancillary hosts, which may harbour disproportionate supplies of virulent pathogen strains.

**Electronic supplementary material:**

The online version of this article (doi:10.1186/s12862-016-0680-6) contains supplementary material, which is available to authorized users.

## Background

The severity of plant, animal and human epidemics depends in part on the interaction between pathogen evolution and host induced selection of virulence traits [1]. Pathogen diversity and evolution is likely influenced by host population size. This is because large host populations can potentially carry large pathogen populations, leading to more opportunities for mutation in the pathogen and thus higher pathogen genetic diversity [2]. Furthermore, the potential for pathogen connectivity through migration is enhanced in large host populations, with transmission ability representing a key factor in pathogen evolution [3, 4].

Most pathogens are able to infect more than one host species, with additional consequences for the evolution of pathogen virulence [5, 6]. Thus, host diversity may strongly influence the diversity of pathogen virulence in an evolutionary arms race between resistance genes in the host and effector genes in the pathogen, on a gene-for-gene basis [7–9]. For example, in the flax rust fungus, *Melampsora lini*, pathogens with broad virulence occur more frequently in highly resistant host populations [10, 11]. Thus a large number of resistance alleles in the host usually select for a large number of effector alleles in the pathogen through balancing or diversifying selection [12–14]*.* At the population level, variation in host resistance is also likely to be of central importance for influencing the patterns of disease incidence and prevalence [15]. It follows, that in order to understand disease dynamics, host diversity is therefore, an important consideration.

Presently, there appear to be few studies investigating the influence of host diversity on pathogen evolution, with most of these studies focused solely on pathogen virulence evolution at traits under selection to the neglect of any consideration of diversity at other loci (but see [16, 17]). Yet, it seems likely that host diversity at loci under selection will also influence the levels of pathogen neutral genetic diversity [18]. For example, higher neutral genetic diversity was found in sexually reproducing populations of the wheat pathogen *Puccinia striiformis* f.sp *tritici* when the alternate host, *Berberis*, was present [16]. In contrast, *Zymoseptoria tritici* exhibits a higher effective population size on cultivated wheat (low genetic diversity), indicating a faster rate of evolution, when compared to its sister species on wild grasses (high genetic diversity) [19]. These contrasting results make it difficult to predict whether host diversity will increase pathogen neutral diversity. Since pathogen neutral genetic diversity is expected to be a good proxy for pathogen evolutionary potential [20], which in turn may affect disease dynamics, it is thus surprising that the influence of host diversity has not been investigated more widely when assessing the importance of factors influencing pathogen neutral genetic diversity.

Although population neutral genetic diversity and virulence (or pathotypes) of pathogens that infect crops have been well documented for a diverse array of pathogens, the influence of susceptible, sympatrically occurring weeds on pathogen diversity or virulence has received remarkably little empirical attention (but see circumstantial support in [21, 22]). This is despite disease management strategies advocating management of weeds because of their potential importance in pathogen evolution [18, 23, 24].

In this study we apply an integrated approach by considering both host diversity and pathogen population size, and their influence on pathogen neutral and selected (virulence, number of pathotypes and diversity at effector loci) genetic diversity. We explore the relationships between pathogen and host genetic diversity and population size, using the well-characterised *Rhynchosporium commune –* barley pathosystem as model. *R. commune* is a haploid fungal pathogen, previously known as *R. secalis* [25] and causing a disease known as barley leaf scald. It is a common pathogen on barley (*Hordeum vulgare* ssp. *spontaneum*), occurring wherever barley is cultivated. The pathogen also infects barley grass, known as the *Hordeum murinum* species complex, consisting of three subspecies *H. murinum*, *H. leporinum* and *H. glaucum* [26].

Within Australia, barley is a major crop resulting in a large census population size (*N*) for the host and pathogen. Barley cultivars are selected for genetic uniformity and typically only 4 to 6 varieties make up the majority (75 %) of barley planted in any one region of Australia, leading to the prediction of a small effective population size (*N*_*e*_) for barley. By contrast, barley grass, *H. murinum*, was an adventitious introduction and is now an ubiquitous grass weed which grows in pockets predominantly within the southern cereal growing regions of Australia, often found growing alongside barley fields. Disease incidence of *R. commune* on barley grass is typically much lower than on barley (C. Linde personal observation), resulting in a small pathogen *N* on barley grass. *Hordeum* species are typically autogamous, although *H. murinum* also show evidence for high levels of outcrossing (despite potential for autogamy) [27, 28]. Thus a relatively high level of genetic diversity is found in *H. murinum* populations [27, 29] with no identical biotypes identified in a small study in Australia [30], leading to the prediction of a large *N*_*e*_ for barley grass*.*

Host immune receptors are mostly encoded by a single dominant resistance (*R*) gene that in turn directs the recognition of a cognate pathogen effector gene [31–33]. The gene-for-gene paradigm implies selection pressure on the pathogen to escape recognition by host resistance genes. In *R. commune*, one such characterized gene-for-gene system is *Rrs1-Nip1* [34]. However, the host resistance conferred by *Rrs1* was overcome in 1956 in California [35] where pathogen virulence evolved via deleted or altered *nip1* genes [34, 36, 37]. Consequently, the use of *Rrs1* as a source of resistance in barley breeding programs has declined. With this decline in the use of cultivars with *Rrs1* resistance, it is expected that selection pressure on *Nip1* will be greater in barley grass, than in cultivated barley, where it is assumed that *Rrs1* occurs naturally in barley grass since wild *Hordeum* is a rich source for resistance genes against *R. commune* [38, 39]. We thus predicted *a priori* that more effector locus alleles will be present in the pathogen population on barley grass.

Given our expectation of large differences in the genetic diversity of its two hosts, we further predicted that *R. commune* will be genetically more diverse on barley grass than on cultivated barley. Coupled with these genetic diversity predictions, we also expected to uncover evidence for higher rates of sexual reproduction within the fungal pathogen on barely grass (see Table 1 for predictions). As in other organisms more generally, sexual reproduction in fungi drives genetic recombination and serves to purge deleterious mutations. Although the occurrence of sexual reproduction for *R. commune* has not yet been observed, it is strongly indicated by population genetic studies [40]. Thus estimating the degree of linkage disequilibrium will be informative.

**Table 1:** Predicted and observed *Rhynchosporium commune* diversity estimates under the contrasting host diversities and pathogen census population size

	Barley			Barley grass		
	Large pathogen pop size	Genetically uniform host		Small pathogen population size	Genetically diverse host	
*R. commune* diversity	Predicted	Predicted	Observed	Predicted	Predicted	Observed
Neutral genetic diversity:						
No. of SSR alleles	**Many**	Few	**Many**	**Few**	Many	**Few**
*Ne*	**Large**	Small	**Large**	**Small**	Large	**Small**
No. of MLGs	**Many**	Few	**Many**	**Few**	Many	**Few**
Mode of reproduction/linkage disequilibrium	-	More asexual/high linkage disequilibrium	More sexual/lower linkage disequilibrium	-	More sexual/low linkage disequilibrium	More asexual/higher linkage disequilibrium
Migration	More from barley to barley grass	**More from barley grass to barley**	**More from barley grass to barley**	More from barley to barley grass	**More from barley grass to barley**	**More from barley grass to barley**
Diversity under selection*:*						
No. of effector alleles	-	**Few**	**Few**	-	**Many**	**Many**
No. of pathotypes	-	**Few**	**Few**	-	**Many**	**Many**
Aggressiveness	-	**Less**	**Less**	-	**More**	**More**

Host dynamics (taken as a given based on literature) for barley is a large acreage/population size with small *N*_*e*_ (few cultivars used), inbred monocultures, assumed few resistance genes/alleles and higher disease (*R. commune*) incidence (large pathogen census population size). Barley grass is a weedy annual which may have a smaller population size than barley (on average less plants), is genetically diverse and thus has a large *Ne*, outcrossing, assumed to have many resistance genes/alleles and has lower disease (*R. commune*) incidence (small pathogen census population size). Observed pathogen genetic diversity and effective population sizes are as observed at neutral loci (SSRs) and traits that may be under selection (effector locus *Nip1*, pathogenicity). Predictions and observations in bold indicate when results confirmed predictions

The first aim of this study is to simultaneously investigate the hypothesis that host genetic diversity and large pathogen *N* leads to high pathogen genetic (neutral and selected) diversity. The second aim is to assess which of these factors is more important for maintaining diversity in the pathogen (see Table 1 for predictions). For this assessment we compare *R. commune* populations from the weedy barley grass host (high genetic diversity, smaller host and associated pathogen *N*) to the monoculture barley crop (low genetic diversity but harbours larger pathogen *N*). For *R. commune* we estimate diversity at both neutral microsatellite loci and for pathogen traits and loci that are predicted to be under selection to answer the following four questions: 1) Does host genetic diversity and/or pathogen *N* affect pathogen neutral genetic diversity and mode of reproduction? 2) Is there more pathogen migration from the weed to the crop, and if so does this enhance the risk of newly evolved virulent strain transfer to barley? 3) Is pathogen genetic diversity under selection related to host genetic diversity? 4) Are pathogen populations from a genetically diverse host more aggressive/virulent than those from a monoculture crop? In our discussion we explore the implications of our improved understanding of the evolutionary processes affecting pathogen diversity. We also predict general outcomes for host-associated pathogen evolution within an evolutionary framework.

## Methods

### Microsatellite analyses

Fourteen neutral microsatellite or simple-sequence-repeat loci (SSR) were used to characterize 170 *R. commune* isolates from barley grass and 150 from barley. Seven populations from barley grass and six from barley were analysed (Additional file 1: Figure S1). Diseased leaves for each population were collected from a 1 × 50 m transect for isolation of *R. commune* (Additional file 2: Appendix B). One isolate per infected leaf was collected 1 m apart to give 12 to 48 isolates collected per transect. SSR loci and analyses followed [41, 42].

### Microsatellite diversity

#### MLG diversity

Isolates with the same combination of SSR alleles at all loci were considered clones or multilocus genotypes (MLGs). The R package Poppr [43] was used to calculate a number of indices describing MLG occurrences and distributions, and linkage disequilibrium indices as set out below. To quantify genotypic diversity by comparing the occurrence and frequency of MLGs among *R. commune* populations, the number of MLGs in each individual and host-associated population and the expected number of MLGs after rarefaction (*eMLG*) was calculated. Furthermore, the Shannon-Wiener index (*H*) of MLG diversity [44] and the evenness index *E.5* estimating the equitability in the distribution of the sampling units and which varies from zero (no evenness) to one (when all MLGs have equal abundance) [45, 46] were estimated. Distribution of MLGs was determined by calculating the occurrence and frequency of recurrent MLGs within and across host populations.

#### Allelic diversity and population structure

Estimates of neutral genetic diversity at the population and host-associated population level were calculated using GenAlEx v6.502 [47, 48]. Estimates included the number of alleles (*N*_*a*_), number of private alleles, Nei’s gene diversity (*H*_*e*_) [49], Shannon’s information index (*I*) [50]. To investigate whether *R. commune* from barley and barley grass belong to divergent genetic pools, population structure was assessed with an AMOVA and principle coordinates analysis (PCoA). Significance for the AMOVA and population differentiation (PhiPt) between pairwise comparisons of population was determined by 999 permutations. Only one individual from each MLG was used in the PCoA analyses. In order to allow a meaningful comparison of genetic diversity within and among host-associated populations we estimated scaled (values ranging from 0 to 1) Shannon diversity (*D’*) levels following Smouse et al. [51].

#### Effective population size and migration

Estimates of the extent of migration between host-associated populations, as well as their respective effective population sizes, was modeled with coalescent simulations in a Bayesian framework as implemented in IMa2 [52, 53]. Earlier population genetic studies have used estimates of population differentiation (such as *F*_*st*_) to indirectly estimate the levels of gene flow. However, the main assumption of migration-drift equilibrium is seldom met in natural systems, thus estimates derived from *F*_*st*_ (or similar estimators) may be flawed [54, 55]. A key advantage of IMa2 is that it does not make these restrictive assumptions. Instead, IMa2 implements an Isolation-with-Migration model which allows estimation of demographic parameters in non-equilibrium systems. One assumption of IMa2 that is not met in the *R. commune* system is free recombination between loci (see Results). However, previous studies have shown that the method is robust to some violation of this assumption, apart from an upward bias in estimations of the ancestral population size [56].

All 14 loci were used to estimate the parameters including the effective population size of each host-associated population and the ancestral population (θ_1_, θ_2_ and θ_A_), together with asymmetrical migration rates between populations (*M*_*1*_ and *M*_*2*_). For details on IMa2 analyses and convergence, see Additional file 2: Appendix B. For conversion of the estimated parameters to demographic values (*N*_*e*_ = effective population size and *Nm* = number of migrants per generation), IMa2 uses the geometric average of mutation rates per locus, multiplied by locus length. Rates were based on substitution rates estimated by Kasuga et al. [57] and assuming one generation per year. The significance of the difference between demographic parameters was evaluated by applying a likelihood ratio tests to obtain probability values, as implemented in the software.

#### Sexual reproduction - linkage disequilibrium

Tests for a random association among SSR alleles of host-associated *R. commune* populations were applied by calculating the index of association (*I*_*A*_) [58] and the standardized index of association ${\overline{r}}_d$ [59], the latter which is independent of the number of loci analysed. These indices estimate the degree of association of alleles at different loci within and among populations compared to that observed in a permutated data set. For physically unlinked loci, a value of zero is expected under random mating, i.e. linkage equilibrium (null model). In contrast, a value significantly larger than zero indicates linkage disequilibrium among loci, which may be achieved by no or infrequent sexual reproduction. *P* values were obtained after 999 permutations in the R package Poppr [43].

### *Nip1* analyses

*Nip1* in *R. commune* from barley (*N* = 191) and barley grass (*N* = 139) were amplified with primers nip1_801F and nip_1558R under conditions described previously [36], before sequencing using an ABI 3130 automated sequencer. Sequences were manually edited before assembly in Sequencher v4.7 and alignment with ClustalW as implemented in Geneious v8 [60], available from http://www.geneious.com. For downstream analyses, the alignment was either trimmed to the coding sequences, or trimmed to the same size but included the signal peptide and introns. All alleles were translated in silico in order to check for consistency in the reading frame and canonical start and stop codons. NIP1 is the product (amino acid sequence) of the effector locus *nip1*. NIP1 type numbering followed and continued from previous studies [36, 37] (GenBank accession numbers = KT340216-KT340474, those of representative sequences of new NIP1 types = KT340207-211, KT340212-213 and KT340215).

In pathogen individuals in which *nip1* is absent (Δ*Nip1*), a positive (disease) interaction is elicited on barley containing *Rrs1* (see Additional file 1: Table S1 for explanations of expected disease reactions)*,* hence the frequency of isolates with Δ*Nip1* provides a strong indication as to whether the pathogen is capable of infecting its host when containing *Rrs1*. Several PCRs were performed to assess the number of isolates with a Δ*Nip1* (Additional file 2: Appendix B).

#### *Nip1* diversity

*Nip1* sequences from barley and barley grass were assessed for basic sequence statistics and diversity measures, in DNaSP v5 [61] and population structure with an AMOVA in GenAlEx. To assess the number of NIP1 types originating from barley and barley grass, existing sequences deposited in GenBank and originating from Australia [36, 37] was added to the analyses.

#### Virulence of NIP1 types

NIP1 types which produced lesions on all three tested cultivars; Atlas 46 (containing *Rrs1*), Atlas (*rrs1*) and Yagan (susceptible check), were deemed virulent on *Rrs1* [36].

### Pathogenicity

#### Isolates and seed types

Genetically unique *R. commune* isolates across the 14 SSR loci were collected from 2005 to 2007 for pathogenicity assays. Eleven isolates from cultivated barley were randomly selected from the Adelaide region and Yorke Peninsula (South Australia) and Horsham (Victoria). Isolates from barley grass were collected from McLaren Vale (8 isolates) South Australia, and one isolate from NSW (Additional file 1: Table S2).

Cross pathogenicity tests were conducted on 20 barley cultivars and 19 barley grass lines (Additional file 1: Table S3). Barley grass mother lines starting from a single barley grass seed head were multiplied in pots in the greenhouse for at least two generations to obtain sufficient seed for pathogenicity trials.

#### Pathogenicity trials

Barley and barley grass lines were grown for one month in 40-cell Hiko trays, one of each barley cultivar and barley grass lines per tray. The position of each seed line was randomly varied across trays. Barley grass seeds were planted 5 days prior to barley seeds to achieve similar development at inoculation, i.e. the three-leaf stage. Each isolate was inoculated onto two trays of plants using ~100 mL spore suspension with 10 *μ*L Tween-20 (Darmstadt, Germany), spraying until runoff. Percentage leaf area infected assessments were made 14 d after inoculation (Additional file 2: Appendix B).

#### Pathogenicity data analyses

Percentage leaf area infected was log transformed (as log10(value + 1)) and treated as the response variable in a linear mixed model analysis using lme4 [62] in R version 3.2.0 [63]. The original host from which isolates was collected, seed type onto which isolates were inoculated and the interaction between host and seed type were specified as fixed factors. Isolates, barley or barley grass cultivar inoculated and the tray replication, were specified as random effects. Post hoc tests were conducted to assess statistical differences in the percentage leaf area infected for isolates from barley and barley grass inoculated on the same hosts. The percentage of barley cultivars and barley grass lines infected by isolates from either barley or barley grass was assessed forstatistical significance with a *t*-test.

## Results

*Rhynchosporium commune* host-associated populations were analysed with 14 SSR loci and an effector locus to assess the relationship of the pathogen’s genetic diversity compared to that of the host and the pathogen’s *N*.

### Microsatellite diversity

#### MLG diversity

In comparison to populations from barley, the populations from barley grass had fewer SSR MLGs (e.g. *eMLG*_barley grass_ = 76, *eMLG*_barley_ = 132), a lower Shannon-Wiener index (*I*_*barley grass*_ = 3.82, *I*_*barley*_ = 4.80) and more even MLG distribution (*E.5*_*barley grass*_ = 0.86, *E.5*_*barley*_ = 0.46). Thus, the pathogen from barley grass had fewer MLGs, which occurred at similar frequencies (Table 2), indicating more asexual reproduction is occurring.

**Table 2:** Microsatellite diversity, multilocus genotypes and indices of linkage disequilibrium for each *Rhynchosporium commune* population

								I_A_ (*P* value)		${\overline{r}}_d$ (*P* value)	
Population	Origin	N	MLG	eMLG	SE	*H*	*E.5*	Not cc^a^	cc^b^	Not cc^a^	cc^b^
Barley grass											
Aldinga	SA	28	3	1.71	0.66	0.31	0.44	5.19 (0.001)	2.91 (0.001)	0.74 (0.001)	0.84 (0.001)
Coriole	SA	12	8	6.83	0.66	1.81	0.68	1.18 (0.008)	0.54 (0.039)	0.15 (0.008)	0.12 (0.016)
Hugo Winery	SA	48	20	6.82	1.26	2.48	0.59	3.19 (0.001)	1.46 (0.001)	0.28 (0.001)	0.25 (0.001)
McLaren Flat	SA	17	7	4.79	0.96	1.40	0.54	3.71 (0.001)	2.45 (0.001)	0.35 (0.001)	0.44 (0.001)
D’Arenburg	SA	20	18	9.53	0.58	2.86	0.95	1.25 (0.001)	0.94 (0.001)	0.10 (0.001)	0.16 (0.001)
Goolaringa	NSW	30	17	7.47	1.16	2.52	0.70	4.92 (0.001)	2.58 (0.001)	0.42 (0.001)	0.45 (0.001)
Horsham Farm	Vic	15	15	10.00	0.00	2.71	1.00	1.11 (0.001)	1.11 (0.001)	0.09 (0.001)	0.09 (0.001)
Barley grass total		**170**	**84**	**76**	**2.00**	**3.82**	**0.86**	**1.72** (0.001)	**1.21** (0.001)	**0.15** (0.001)	**0.10** (0.001)
Barley											
Paskeville	SA	20	20	10.00	0.00	3.00	1.00	0.31 (0.019)	0.33 (0.014)	0.03 (0.019)	0.06 (0.014)
Port Clinton	SA	6	5	5.00	0.00	1.56	0.93	4.02 (0.001)	3.00 (0.001)	0.46 (0.001)	0.63 (0.001)
Brentwood	SA	33	30	9.74	0.47	3.37	0.99	1.32 (0.001)	0.70 (0.001)	0.10 (0.001)	0.12 (0.001)
South Australia	SA	35	31	9.54	0.66	3.36	0.87	1.09 (0.001)	0.66 (0.001)	0.09 (0.001)	0.11 (0.001)
Werribee	Vic	15	14	9.57	0.49	2.62	0.97	1.87 (0.001)	1.06 (0.001)	0.16 (0.001)	0.18 (0.001)
Horsham	Vic	41	37	9.74	0.49	3.57	0.93	1.27 (0.001)	0.74 (0.001)	0.10 (0.001)	0.13 (0.001)
Barley total		**150**	**132**	**132**	**0.00**	**4.80**	**0.46**	**0.83** (0.001)	**0.60** (0.001)	**0.07** (0.001)	**0.05** (0.001)
Total		**320**	**216**	**9.53**	**0.70**	**4.98**	**0.45**	**1.16** (0.001)	**0.64** (0.001)	**0.09** (0.001)	**0.11** (0.001)

*SA* South Australia, *NSW* New South Wales, *Vic* Victoria

*N* number of *R. commune* isolates analysed

*eMLG* The number of expected MLG at the smallest sample size based on rarefaction [89] with standard error (SE)

*H* Shannon-Wiener Index of MLG diversity [46]

*E.5* Evenness, ie equitability in the distribution of the sampling units [45, 46]

Linkage disequilibrium indices ${\overline{r}}_d$ [59] and the index of association (I_A_) [58]

^a^Not clone-corrected data set

^b^Clone corrected data set

#### Allelic diversity and population structure

Neutral genetic diversity as analysed via the 14 SSR loci showed that when compared with barley, populations of the pathogen from barley grass had lower *H*_*e*_, fewer alleles and lower Shannon information indices (Additional file 1: Table S4). In combined host-associated populations the number of alleles, number of private alleles, Nei’s gene diversity and Simpson’s information index were all higher in the barley *R. commune* populations (Additional file 1: Table S5). In the AMOVA, only 9 % (*P* = 0.001) of the genetic diversity was attributable to host differences (PhiRT = 0.09), with most diversity (69 %) attributable to differences among individuals within populations (PhiPT = 0.309) (Additional file 1: Table S6). Population differentiation between host-associated populations was low (PhiPt = 0.064, *P* = 0.001). Scaled Shannon diversity was higher among pathogen populations from barley grass than barley (*δ′*_*barley grass*_ = 0.414, *δ′*_*barley*_ = 0.331), while higher diversity was found within barley associated pathogen populations than in barley grass (*β′*_*barley*_ = 0.734, *β′*_*barley grass*_ *=* 0.435) (Additional file 1: Table S7). Principle component analyses revealed a single overlapping MLG cluster with host-associated MLGs tending to cluster at opposite ends (Additional file 1: Figure S2). Axis 1 and 2 of the PCoA accounted for 9.4 and 7.1 % of the total genetic variability, respectively, indicating a lack of strong structure.

#### Effective population size and migration

The IMa2 marginal posterior distributions for the scaled *N*_*e*_ of *R. commune* from barley was higher than that for barley grass, but with overlapping 95 % highest posterior density intervals. Effective population size of the ancestral population was orders of magnitude (>100×) higher than the pathogen *N*_*e*_ from either barley or barley grass. Although estimates of ancestral populations may in part be inflated due to a violation of the no linkage among loci assumption in IMa2 [56], the large orders of magnitude difference observed here cannot be solely due this effect. Rather the results indicate significant population contraction, likely as a result of genetic drift in the founder *R. commune* populations in Australia. The analysis further indicates migration of *R. commune* between both host populations, with the extent of migration (*2 Nm*) from barley grass to the barley population significantly (LRR test = 4.296; *P* < 0.05) higher than in the reverse direction (Table 3).

**Table 3:** Bayesian estimates of scaled demographic parameters and 95 % highest posterior density (HPD) intervals of *Rhynchosporium commune* as estimated under an isolation with migration model (IMa2) using 14 microsatellite loci

Parameter	Mode	HPD95L	HPD95H
Effective population size (*2N*_*i*_ *μ)*			
Barley	115.3	30.34	285.2
Barley grass	42.48	18.21	176
Ancestral	12131	11075	23451
Population migration rate (*2 Nm)*			
From barley to barley grass	0.896	0.535	2.229
From barley grass to barley	4.164	2.939	7.840

Parameters are estimated from the peak location of the estimated probability densities. Population size and migration parameters are scaled by the mutation rate *μ*; N_i_ the effective size of population i; *μ*, the mutation rate; *m*, the migration rate per generation with one generation per year

#### Sexual reproduction - linkage disequilibrium

Both ${\overline{r}}_d$ and *I*_*A*_ indicated significant levels of non-random association of alleles across all populations within a host and across the combined host-associated populations. Levels of linkage disequilibrium were highest in pathogen populations from barley grass, especially of non-clone corrected populations as expected given the large number of clones identified in those populations (Table 2).

### *Nip1* analyses

#### *Nip1* diversity

In total, 12 NIP1 protein types were found to occur in Australia. Six NIP1 types were found among 191 isolates from barley and eight types among 139 isolates from barley grass, with two types shared among the host populations. Isolates lacking *nip1* (ΔNIP1) are pathogenic on barley cultivars containing *Rrs1*. The proportion of isolates with ΔNIP1 varied from only four of the tested 274 isolates (1.45 %) from barley, to 44 of the tested 192 isolates (22.92 %) from barley grass (Table 4). *Nip1* nucleotide diversity showed similar values for isolates from barley and barley grass, although nine polymorphic sites were found among isolates from barley grass compared to only four among isolates from barley (Additional file 1: Table S8). An AMOVA revealed a similar pattern of population structure at *nip1* as observed for the SSRs (Additional file 1: Table S6).

**Table 4:** NIP1 types of *Rhynchosporium commune* from barley and barley grass in Australia. Virulence is as measured on the barley cultivar Atlas46, which contains the resistance gene *Rrs1*

		Number of isolates		
NIP1 type	No of isolates	Barley	Barley grass	Virulence on *Rrs1*
1	30	28	2	-
2	232	155	77	-
3	2	-	2	+
9	1	1	-	-
14	51	-	51	-
26	1	1	-	+
27	3	-	3	+
28	5	5	-	+
29	1	-	1	-
30	1	1	-	-
31	2	-	2	+
32	1	-	1	-
SUM	330	191	139	
Δ*Nip1*		4/274	44/192	+

#### Virulence of NIP1 types

Three NIP1 types (3, 27 and 31) from barley grass and two from barley (26 and 28, the latter lacking a signal peptide), as well as isolates with ΔNIP1, were virulent on all three barley cultivars tested, hence these confer virulence to *Rrs1* containing cultivars (Table 4).

### Pathogenicity

**Fig. 1:**
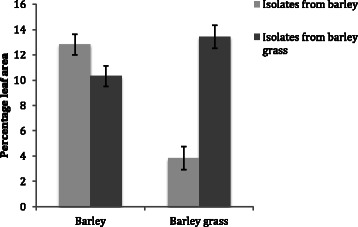
The mean percentage leaf area infected (±SE) with *Rhyncosprorium commune* isolates from barley and barley grass inoculated onto the same two hosts

The linear mixed model analysis of the factors affecting the percentage leaf area covered by lesions showed a significant (*P* < 0.001) effect of host (from which the pathogen was collected) and the interaction between host and seed type (on which isolates were inoculated) (Table 5). The percentage leaf area covered by lesions on barley was significantly (*P* = 0.031) higher for isolates from barley (mean = 12.83 %) than for isolates from barley grass (mean = 10.32 %). In contrast, isolates from barley caused significantly (*P* < 0.001) less disease on barley grass (mean = 3.85 %) than isolates from barley grass (mean = 13.42 %) (Fig. 1). Isolates from barley grass also infected significantly more barley cultivars (91.1 % vs. 87.7 %; *P* = 0.011) and barley grass lines (95.5 % vs. 79.5 %; *P* < 0.001) than isolates from barley. While these results show strong evidence for host adaptation, they nonetheless also confirm that isolates from barley grass are capable of infection on barley grass. Furthermore, such isolates also caused larger lesions on barley.

**Table 5:** Linear mixed model assessing the effects of host and seed type on the percentage leaf area covered by lesions

Term	Estimate	SE	*t*	*P*
Intercept	0.658	0.359	1.829	0.067
Host B	−0.403	0.063	−6.351	<0.001
Seed type B	0.004	0.066	0.059	0.952
Host B: Seed type B	0.407	0.037	11.030	<0.001

Host = host from which pathogen was isolated; Seed type = either barley (B) or barley grass (BG) seed used for seedling growth onto which inoculations were performed

## Discussion

In this detailed genetic study we investigated the often-overlooked hypothesis that host genetic diversity and pathogen population size affects pathogen diversity. It is well-known that variation in the host phenotype affects virulence evolution in the pathogen [64]. However, studies that simultaneously evaluate host diversity and pathogen population size at neutral and selected loci in order to explore the link between fungal pathogen diversity and virulence appear to be absent. We predicted that pathogen neutral genetic diversity will increase with large pathogen *N* AND host diversity, whereas pathogen diversity under selection will increase with host diversity. This prediction was broadly supported, with the exception that pathogen neutral genetic diversity was found to increase with pathogen *N*. We also discovered strong evidence that pathogen migration from the diverse host (barley grass) is high, providing a mechanism for the effective transmission of virulence from weeds to the crop. Below we explore these findings and their implications in more detail.

### Does host genetic diversity and/or pathogen *N* affect pathogen neutral genetic diversity and mode of reproduction?

Predicting whether pathogen neutral genetic diversity, and thus *N*_*e*_ will change with host genetic diversity and/or host/pathogen *N*, is not necessarily straightforward. The number of alleles/genotypes maintained by mutation is expected to increase strongly with population size. Furthermore, irrespective of *N*, diverse host populations are predicted to select for diverse pathogen populations, likely via enhanced sexual reproduction in the pathogen, such as the case of *Puccinia graminis* on its alternate host [65, 66] and selection favouring novel mutations such as in rusts on wild oats in Australia [21, 22].

Whether or not host diversity overrides the importance of mutation in large populations, is an open question. The interplay between *N* and host diversity becomes particularly important when one considers weeds as a potential ancillary host of crop pathogens. This is because weeds are typically genetically more diverse than crops, but will often have smaller population sizes or sustain lower disease incidence. The possibility of variation in the degree of sexual reproduction between hosts may add a further layer of complexity.

Most fungi can reproduce both asexually and sexually, with fungal populations of a species with high rates of sexual reproduction typically being genetically more diverse than populations with less sexual reproduction. In fungal plant pathogens a key advantage of sexual reproduction is that it can combine virulences from two individuals into the same genetic background, thereby accelerating adaptation to novel combinations of resistance genes in the host. Meanwhile, the pathogen’s capacity for asexual reproduction can enable rapid propagation, while keeping well-adapted gene combinations together. In the present study of *R. commune* from barley, all neutral genetic diversity estimates based on the 14 SSR loci, including the number of MLGs, number of alleles, Nei’s gene diversity, Shannon’s information index and the scaled within population Shannon diversity, were higher than for populations from barley grass. This suggests lower levels of clonal reproduction in the pathogen on barley, in contrast to the expectation that pathogen neutral genetic diversity should be higher on the more diverse host. This is an unexpected finding since sex in fungi is often triggered by stress [67], which is predicted to be higher in non-cultivated host populations.

In an extension of genetic diversity estimates, the IMa2 *N*_*e*_ estimate for the pathogen population from barley was approximately twice as large as that from barley grass, although we note some overlap of posterior density intervals. Nevertheless, the findings suggest that the larger population size (acreage) of barley is accompanied by an associated larger pathogen *N* with a more diverse pathogen population. Therefore, in our study system, host/pathogen *N* seems to be more important than host diversity for maintaining large *N*_*e*_ and higher neutral genetic diversity of the pathogen. That is, higher pathogen neutral genetic diversity is correlated with a higher host/pathogen *N*, not higher host diversity, at least at loci evolving neutrally. This finding may reflect the reduced risk of rare allele loss in larger pathogen populations [68].

Although we predicted differences in the degree of pathogen sexual reproduction on barley and barley grass, both host-associated populations were in linkage disequilibrium, suggesting both are predominantly asexual. This finding is consistent with the observation that the sexual state has never been observed for *R. commune*. However, other lines of evidence suggest some sexual reproduction must occur in the pathogen, but its frequency appears to be insufficient to remove the strong signal of disequilibrium generated by recent founder events and bottlenecks [42].

Despite the common finding of disequilibrium in *R. commune* on both barley and barley grass, we found fewer MLGs and these were also distributed more evenly and at higher linkage disequilibrium in barley grass populations. This finding contradicts our prediction that the pathogen should reproduce sexually (have lower linkage disequilibrium) more frequently on barley grass than on barley. It is evident that studies that fail to combine careful and simultaneous consideration of both host genetic diversity and pathogen *N* may draw misleading conclusions.

### Is there more pathogen migration from the weed to the crop?

A key factor for virulence evolution is the effectiveness of between-host transmission [6, 69–71]. It is therefore important to establish whether between-host transmission is occurring, in order to assess the importance of alternate hosts on pathogen evolution. Few studies have shown ongoing pathogen migration between wild and cultivated host plants [72, 73]. Our estimates of migration rates indicated there is no transmission barrier between the two hosts that would prevent newly evolved virulence types selected on barley grass from migrating to barley. In fact, the estimated migration rates of the pathogen from barley grass into the barley pathogen populations were significantly (*P* < 0.05) higher. These higher migration rates might be facilitated by a higher number of resistance genes in *H. murinum* [39], which block more migrant pathogen isolates from barley establishing on barley grass, than in the reverse migration. This further suggests that newly evolved virulence on the barley grass can be rapidly transmitted to barley-infecting populations, posing a significant threat to durability of disease resistance genes bred into barley cultivars.

### Is genetic diversity under selection at an effector locus related to host genetic diversity?

The potential importance of frequency-dependent selection in maintaining genetic diversity within host-pathogen interactions is well recognized [74, 75]. However, while it is acknowledged in theory that weedy hosts could play a role in the evolution of plant pathogens [18, 24, 39, 76], investigations are often limited to simply quantifying the number of pathotypes present. Isolates from barley grass in this study exhibited slightly higher *nip1* nucleotide diversity, more NIP1 amino acid types and harboured more virulent NIP1 types. Strong evidence for selection on NIP1 composition was also found in the higher frequency of Δ*NIP* (Δ*Nip1* = where *nip1* is absent) in isolates from barley grass. Isolates with Δ*NIP1* are able to escape host resistance conferred by *Rrs1* [34, 36]*,* indicating a high prevalence of *Rrs1* in barley grass is selecting for infectious strains. Our results are therefore consistent with the expectation of frequency-dependent selection [74, 75] on the genetically more diverse weedy host driving increased diversity of the pathogen populations, despite smaller pathogen *N*.

### Are pathogen populations from a genetically diverse host more aggressive/virulent than those from a monoculture crop?

More than 40 years ago, Ali and Boyd [77] suggested that weedy barley grass could have an important influence on the epidemiology of *R. commune* on cultivated barley. Since then, other studies have reported that isolates from barley grass were highly variable for pathogenicity [30, 39, 78] or display novel virulences [79]. Brown [78] found that in a comparison of 182 isolates from barley grass and 94 from barley, isolates from barley grass were significantly more virulent (could infect more cultivars) and had more pathotypes (19 in barley grass vs. 5 in cultivated barley isolates). Similarly, the present study found that despite evidence for host adaptation (isolates from barley tend to be more aggressive on barley, and the converse on barley grass), isolates from barley grass infected significantly more barley cultivars (91.1 % vs. 87.7 %; *P* = 0.011) and overall they were more aggressive than isolates from barley. Weeds, or genetically diverse hosts, as a source of virulence is often assumed but is rarely shown empirically, with a few notable exceptions [21, 22, 65, 73]. Combined with the evidence for *R. commune* migration between hosts as shown here and in [72], it is evident that weedy or wild hosts could play a major role in pathogen evolution.

### Implications for pathogen evolution

Our study of barley/barley grass *R. commune* populations confirm that there is indeed a relationship among host and pathogen diversity and population sizes, but that the direction of the relationship can vary between neutral loci/traits to those under selection. In this case, increased pathogen diversity of effector alleles at *nip1*, but not alleles at neutral loci, was found on the genetically more diverse host, barley grass. This pattern of increased diversity at *nip1* is consistent with an arms race between host and pathogen. Indeed, similar findings have been found for parasites [9, 80], bacteria [81] and rust pathogens on crops [16, 65] and wild hosts [10, 11], where pathogen race diversity is higher on diverse or alternate hosts where sexual reproduction occurs. An exception is found in the poplar rust pathogen *Melampsora larici-populina* with fewer pathotypes in wild than in cultivated populations (although some results were inconclusive), likely because of complex resistances bred into cultivated poplars [17, 82] and ongoing gene flow [73]. The contrasting findings that the neutral loci of the *R. commune* pathogen did not exhibit more diversity on the genetically more diverse host, suggest that pathogen *N* is a better predictor of the patterns of pathogen neutral genetic diversity. Similarly *Zymoseptoria tritici* shows evidence for faster genomic evolution and a higher effective population size on cultivated wheat compared to its sister species on wild grasses [19]. The discrepancy between effector alleles and neutral genetic diversity may also be attributed to the rapid rate at which virulence evolves on cultivated hosts due to the high selection pressure exerted by widely deployed resistance R-genes [20].

### Practical implications for agriculture

Agricultural mechanisation has led to an ever increase in crop acreages with serious consequences for disease risk. In this study of *R. commune,* the large acreage of barley and associated large pathogen *N* was linked with increased pathogen neutral diversity, despite the lower genetic diversity of the host. It follows that host/pathogen population sizes need to be kept small to reduce the risk of accelerated adaptive evolution in the pathogen [20]. One strategy to achieve this is to employ various alternatives to crop monocultures such as intercropping (mixtures of different crop species) or the use of multiline cultivars or varietal mixtures (crop plants that differ in resistance specificities). Applying the strategy of host heterogeneity (eg. multilines or cultivar mixes) is known to negatively affect the pathogen’s ability to survive, reproduce and compete in several systems [83].

Ensuring host heterogeneity is also recommended to extend the life of resistant cultivars by retarding the rate of pathogen evolution [84, 85]. However, a potential risk of this strategy is that extensive use of host heterogeneity could lead to the development of complex pathogen races [86]. Indeed, in our case study of barley grass as a heterogeneous host, our findings of more pathotypes and higher virulence diversity compared with the pathogen on barley highlights this risk. To retard pathogen evolution, it is thus important to combine and rotate as many disease control and management strategies in space and time as possible [20, 23, 24, 87, 88].

Management of disease on crops must also include management of weedy ancillary hosts, which, as evident in this study, can harbour disproportionate supplies of virulent pathogen strains. However, it will be particularly challenging to manage ubiquitous weeds or wild hosts for this purpose. Applying herbicides adjacent to cultivated hosts is a feasible starting point, but may not prevent pathogen transmission from further a field. This is especially true for airborne pathogens or those where migration is assisted by human-mediated dispersal of infected plant material.

## Conclusions

Resistance genes typically have a short life time because pathogens evolve virulence [20]. This arms race between hosts and pathogens depend mostly on interactions between their genetic diversity, population size, transmission ability and host composition. Although it has long been implied that weeds may play a key part in these interactions, empirical evidence demonstrating this is rare. The findings of this study reinforce the critical role weeds play in the evolution of pathogen virulence.

### Availability of data and materials

The nucleic acid sequences of *nip1* generated for this article are available in the GenBank repository with accession numbers of nip1 = KT340216-KT340474, those of representative sequences of new NIP1 types = KT340207-211, KT340212-213 and KT340215.

## Additional files

Additional file 1: Appendix A. Supporting Information. (DOCX 603 kb) Additional file 2: Appendix B. Supplementary information. (DOCX 35 kb)
