# Supplementary material for: Weeds, as ancillary hosts, pose disproportionate risk for virulent pathogen transfer to crops
Source: BMC Evol Biol. 2016 May 12;16:101. doi: 10.1186/s12862-016-0680-6 (PMC4866072; doi:10.1186/s12862-016-0680-6)
Supplement: Additional file 1: — Appendix A. Supporting Information. (DOCX 603 kb) [file 12862_2016_680_MOESM1_ESM.docx]

**Supporting Information**

**Figure S1**. Map of *Rhynchosporium commune* and barley grass seed collections in Australia.

**Figure S2.** Scatter plot of the first two components of the principle coordinates analyses of the 216 different multilocus genotypes of *Rhynchosporium commune* sampled from barley and barley grass in Australia.

**Table S1.** The expected disease reaction between homozygous host and pathogen genotypes in a single-locus gene-for-gene interaction

|  | **Host genotype** | |
| --- | --- | --- |
| **Pathogen genotype** | ***Rrs1*** | ***rrs1*** |
|  |  |  |
| ***Nip1*** | No disease | Disease |
| ***nip1*** | Disease | Disease |
| **Δ*Nip1*** | Disease | Disease |

*Rrs1* is a dominant resistance gene in barley conferring resistance to *R. commune* and *rrs1* is a recessive resistance gene conferring susceptibility. *Nip1* is a dominant *R. commune* effector locus conferring avirulence and *nip1* is a recessive (changed, eg. having mutations that alter the effector protein structure) effector locus. The latter confers virulence on *Rrs1* containing host lines*.* When *Nip1* is absent due to a deletion, ie Δ*Nip1*, virulence is conferred *Rrs1* containing host lines.**Table S2.** *Rhynchosporium commune* isolates used in pathogenicity trials

| Isolate | Host | | Origin |  |
| --- | --- | --- | --- | --- |
| Cor10.1 | Barley grass | | McLaren Vale, South Australia |  |
| Cor12.1 | Barley grass | | McLaren Vale, South Australia |  |
| DAr2-11.1.1 | Barley grass | | McLaren Vale, South Australia |  |
| DAr2-3.4 | Barley grass | | McLaren Vale, South Australia |  |
| Hugo2-12.3.2 | Barley grass | | McLaren Vale, South Australia |  |
| Hugo2-6.2.2 | Barley grass | | McLaren Vale, South Australia |  |
| McC3.3 | Barley grass | | McLaren Vale, South Australia |  |
| McC5.1 | Barley grass | | McLaren Vale, South Australia |  |
| Gool28.2 | Barley grass | Goolaringa, NSW | |  |
| BrGr1.2.1 | Barley | Yorke Peninsula, South Australia | |  |
| PasG2_5.1.1 | Barley | Yorke Peninsula, South Australia | |  |
| SA1192 | Barley | Adelaide region, South Australia | |  |
| SA1201 | Barley | Adelaide region, South Australia | |  |
| SA1225 | Barley | Adelaide region, South Australia | |  |
| SA1248 | Barley | Adelaide region, South Australia | |  |
| SA1252 | Barley | Adelaide region, South Australia | |  |
| SA1260 | Barley | Adelaide region, South Australia | |  |
| SA1263 | Barley | Adelaide region, South Australia | |  |
| HoC9.2 | Barley | Horsham, Victoria | |  |
| HoA1 | Barley | Horsham, Victoria | |  |

**Table S3.** Barley and barley grass lines used in pathogenicity trials

|  | **Line** | **Origin** | |  | |
| --- | --- | --- | --- | --- | --- |
| **Barley** |  |  | | |  |
| 1 | Abyssinian |  | | |  |
| 2 | Atlas |  | | |  |
| 3 | Atlas46 |  | | |  |
| 4 | Barque |  | | |  |
| 5 | Brier |  | | |  |
| 6 | Chieftain |  | | |  |
| 7 | Dash |  | | |  |
| 8 | Franklin |  | | |  |
| 9 | Gairdner |  | | |  |
| 10 | Icarda 4 |  | | |  |
| 11 | Jet |  | | |  |
| 12 | Modoc-Calif |  | | |  |
| 13 | Nigrinudum |  | | |  |
| 14 | O'Connor |  | | |  |
| 15 | Osiris |  | | |  |
| 16 | Skiff |  | | |  |
| 17 | Sloop |  | | |  |
| 18 | Turk |  | | |  |
| 19 | VB 9104 |  | | |  |
| 20 | Yagan |  | | |  |
|  |  |  | | |  |
| **Barley grass** |  |  | | |  |
| 1 | Ald2 | McLaren Vale, South Australia | | |  |
| 2 | Ald5 | McLaren Vale, South Australia | | |  |
| 3 | Cor2 | McLaren Vale, South Australia | | |  |
| 4 | Cor5 | McLaren Vale, South Australia | | |  |
| 5 | D'Ar1 | McLaren Vale, South Australia | | |  |
| 6 | D'Ar4 | McLaren Vale, South Australia | | |  |
| 7 | Hugo1 | McLaren Vale, South Australia | | |  |
| 8 | Hugo2 | McLaren Vale, South Australia | | |  |
| 9 | UCE1 | Canberra, ACT | | |  |
| 10 | CSEAC2.1 | Canberra, ACT | | |  |
| 11 | CSEPS1.2 | Canberra, ACT | | |  |
| 12 | CSEPS1.3 | Canberra, ACT | | |  |
| 13 | CSEPS3.1 | Canberra, ACT | | |  |
| 14 | CSEPS5 | Canberra, ACT | | |  |
| 15 | CSEPS6 | Canberra, ACT | | |  |
| 16 | CSEPS7.1 | Canberra, ACT | | |  |
| 17 | Scriv2 | Canberra, ACT | | |  |
| 18 | Sta1 | Stawell, Victoria | | |  |
| 19 | BHOZ1 | Barwon Heads, Victoria | | |  |
|  |  |  |  | |  |

**Table S4.** Microsatellite diversity of *Rhynchosporium* populations

|  |  |  |  |  |  |  |
| --- | --- | --- | --- | --- | --- | --- |
| Population | *H_e_* | SE | Na | SE | *I* | SE |
| From barley grass:  Aldinga | 0.039 | 0.009 | 1.571 | 0.137 | 0.088 | 0.021 |
| Coriole | 0.190 | 0.053 | 2.000 | 0.296 | 0.345 | 0.099 |
| D’Arenburg | 0.480 | 0.062 | 3.643 | 0.401 | 0.904 | 0.128 |
| Goolaringa | 0.339 | 0.048 | 4.071 | 0.486 | 0.683 | 0.096 |
| Horsham Farm | 0.496 | 0.052 | 3.214 | 0.334 | 0.870 | 0.104 |
| Hugo Winery | 0.334 | 0.069 | 3.071 | 0.305 | 0.585 | 0.112 |
| McLaren Flat | 0.193 | 0.042 | 2.286 | 0.244 | 0.368 | 0.079 |
| From barley:  Paskeville | 0.609 | 0.056 | 5.071 | 0.412 | 1.231 | 0.118 |
| Port Clinton | 0.377 | 0.073 | 2.143 | 0.294 | 0.600 | 0.127 |
| South Australia | 0.627 | 0.058 | 6.143 | 0.811 | 1.313 | 0.147 |
| Werribee | 0.485 | 0.067 | 3.643 | 0.325 | 0.915 | 0.126 |
| Brentwood | 0.622 | 0.061 | 5.643 | 0.498 | 1.281 | 0.132 |
| Horsham | 0.627 | 0.056 | 6.571 | 0.600 | 1.335 | 0.133 |
| **Mean** | **0.417** | **0.020** | 3.775 | 0.164 | 0.809 | 0.042 |

*H_e_* = Nei’s gene diversity ([Nei, 1978](#_ENREF_49)), *I* = Shannon’s information index ([Brown & Weir, 1983](#_ENREF_8)).

**Table S5.** Diversities of *Rhynchosporium* *commune* populations from barley grass and barley

| ***R. commune* host**  **population** | **Number of alleles (*N_a_)***  **± SE** | **Number of private alleles ± SE** | ***H_e_* ± SE** | ***I* ± SE** |
| --- | --- | --- | --- | --- |
| Barley grass | 7.79 ± 0.84 | 2.07 ± 0.41 | 0.47 ± 0.07 | 0.97 ± 0.13 |
|  |  |  |  |  |
| Barley | 11.14 ±1.06 | 5.43 ± 0.68 | 0.67 ± 0.06 | 1.54 ± 0.14 |

*H_e_* = Nei’s gene diversity ([Nei, 1978](#_ENREF_49)), *I* = Shannon’s information index ([Brown & Weir, 1983](#_ENREF_8)).

**Table S6.** Outcomes of hierarchical Analyses of Molecular Variance partitioning of *Rhynchosporium commune* SSR and *Nip1* data among and within hosts and populations. *P*-value estimates are based on 999 permutations. df = degrees of freedom, SS = sum of squares, MS = mean squared deviations

| **Source** | **df** | **SS** | | **MS** | | **Estimated variance** | **Percentage variance** | **AMOVA statistics** | ***P*** |
| --- | --- | --- | --- | --- | --- | --- | --- | --- | --- |
| **SSR** |  |  | |  | |  |  |  |  |
| Among host groups | 1 | 98.142 | | 98.142 | | 0.413 | 9% | PhiRT = 0.09 | 0.001 |
| Within hosts | 12 | 300.982 | | 25.082 | | 1.004 | 22% | PhiPR = 0.24 | 0.001 |
| Within populations | 306 | 971.755 | | 3.176 | | 3.176 | 69% | PhiPT = 0.309 | 0.001 |
|  |  |  | |  | |  |  |  |  |
| ***Nip1*** |  |  | |  | |  |  |  |  |
| Among host groups | 1 | 19.323 | | 19.323 | | 0.093 | 6% | PhiRT = 0.063 | 0.001 |
| Within hosts | 25 | 84.154 | | 3.366 | | 0.184 | 12% | PhiPR = 0.133 | 0.002 |
| Within populations | 303 | 363.056 | | 1.198 | | 1.198 | 81% | PhiPT = 0.187 | 0.001 |
|  |  | |  | |  |  |  |  |  |

**Table S7.** Scaled Shannon diversity within and among *Rhyncosporium commune* populations from barley (B) and barley grass (BG).

| Host-associated *R. commune* populations | Information component | [0,1]-Scaled Shannon diversity (*D*') | *P*-value |
| --- | --- | --- | --- |
| *R. commune* from B | Among populations | δ*'*  = 0.331 | 0.001 |
|  | Within populations | *β'* = 0.734 |  |
|  | Total | *τ'* = 0.792 |  |
|  |  |  |  |
| *R. commune* from BG | Among populations | δ*'* = 0.414 | 0.001 |
|  | Within populations | *β' =* 0.435 |  |
|  | Total | *τ'* = 0.625 |  |

**Table S8.** Nucleotide diversities in *nip1* in *Rhynchosporium commune* populations associated with barley and barley grass in Australia

|  | *R. commune* associated with | |
| --- | --- | --- |
|  | Barley | Barley grass |
| Sample size | 191 | 139 |
| No. of polymorphic sites | 4 | 9 |
| Avg. number of nucleotide differences (*k*) | 0.787 | 0.744 |
| Nucleotide diversity (*p_i_*) | 0.0043 | 0.0041 |

As measured in 183 coding nucleotides, excluding indels.
