# Supplementary material for: Weeds, as ancillary hosts, pose disproportionate risk for virulent pathogen transfer to crops
Source: BMC Evol Biol. 2016 May 12;16:101. doi: 10.1186/s12862-016-0680-6 (PMC4866072; doi:10.1186/s12862-016-0680-6)
Supplement: Additional file 2: — Appendix B. Supplementary information. (DOCX 35 kb) [file 12862_2016_680_MOESM2_ESM.docx]

**Supplementary information**

**Appendix B**

By extension the Red Queen Hypothesis [[1](#_ENREF_1), [2](#_ENREF_2)] predicts that pathogens infecting sexual hosts, such as outcrossing weeds, should have higher rates of sexual reproduction to enhance genetic diversity, than those pathogens infecting asexual hosts, such as self-pollinating crops. This is predicted because the sexual recombination of resistance genes drives negative frequency-dependent selection on the corresponding virulence genes of pathogens. However, the strength of this interaction can be difficult to predict, since it depends on the interplay between host population size and genetic diversity. Despite its clear importance, simultaneously teasing apart the role of population size and genetic diversity in both hosts and pathogens, in order to improve our understanding of how these factors interact with pathogen evolution, has received surprisingly little research attention.

**Materials and Methods**

**Microsatellite analyses**

Fourteen neutral microsatellite (SSR) loci were used to characterize 170 *R. commune* isolates from barley grass and 150 from barley. Seven populations from barley grass and six from barley were analysed. Diseased leaves for each population were collected from a 1 x 50 m transect for isolation of *R. commune*. Five barley grass populations were collected in the McLaren Vale area (South Australia), and one each from Horsham (Victoria) and Goolaringa (NSW). Barley populations were sampled from Yorke Peninsula, South Australia (3 populations), a wider South Australian population (Adelaide region), Werribee (Victoria) and Horsham (Victoria) (Figure S1). Populations from barley and barley grass in South Australia were sourced as close as possible to each other to enable meaningful population diversity comparisons. Unfortunately Australia experienced a number of exceptionally dry years during the sampling period rendering the disease present in only a few areas on barley grass. Isolates from barley were collected within a 400 km radius from barley grass populations, but most often were within 100-150 km. An exception is the Goolaringa population from barley grass, which is approximately a 1000 km away, and the Werribee population from barley, which is approximately 600 km away from most of the populations analysed in this study. One isolate per infected leaf was collected 1 m apart to give 12 to 48 isolates collected per transect. Microsatellite loci and analyses followed [[3](#_ENREF_3), [4](#_ENREF_4)].

***Nip1* analyses**

Pathogen individuals in which *nip1* is absent (Δ*Nip1*), a positive (disease) interaction is elicited on barley containing *Rrs1* (see Table S1 for explanations of expected disease reactions)*,* hence the frequency of isolates with Δ*Nip1* provides a strong indication as to whether the pathogen is capable of infecting its host when containing *Rrs1*. To assess the number of isolates with a Δ*Nip1*, several PCRs were performed after DNA concentrations were adjusted to ~20ng per reaction, with primers obtained from Schürch *et al* [[5](#_ENREF_5)] or designed in this study. All isolates (278 from barley and 236 from barley grass) were initially subjected to PCR using primers nip1_1117F [[5](#_ENREF_5)] and nip1_1431R (5'TGCACCGATGTATTTCAGTGTTTAGC). Isolates that did not give a strong positive amplicon, were then subjected to PCR with several primer combinations to confirm *nip1* deletion. These included nip1_1117F and nip1_1200R [[5](#_ENREF_5)], giving a 119bp amplicon starting 3’ to the signal peptide sequence and ending 5’ to the intron. Nip1_1117F and nip1_1200R (5'AGCCATTGCCACCTTCAC), nip1_1021F (5'ATGAAATTCCTCGTACTGCCTCTCTC) and nip1_1200R [[5](#_ENREF_5)] giving a 333bp amplicon starting at the initiation codon and ending 19bp 3’ to the termination codon, and, nip1_925F (5'ATCACATACCAGTCTTTGATTCC) and nip1_1431R giving a 553bp amplicon starting 92bp 5’ to the initiation codon and ending 147bp 3’ to the termination codon. PCR reactions consisted of 1x Mango Taq colourless PCR buffer (Bioline), 2.5mM MgCl2, 0.125mM dNTPs, 0.5mg/mL BSA (Sigma A-7906), 0.5mM each primer, 0.75U Mango Taq (Bioline). Thermal cycling parameters were 95°C 2’30” then 35x (94°C 20”, 54°C 30”, 72°C 30”). Mating type primers [[6](#_ENREF_6)] were used as a positive control for these samples to confirm DNA quality.

**Genetic data analyses**

**Effective population size and migration:** While more than two populations can be analysed simultaneously in IMa2, for robust conclusions this requires more loci than was available in this study [[7](#_ENREF_7)], as convergence was difficult to achieve. Thus, here we restricted our analyses to combined sets of population samples grouped into the two host-associated populations, which were also our main focus. All 14 loci were used to estimate parameters including the effective population size of each host-associated population and the ancestral population (θ_1_, θ_2_ and θ_A_), together with asymmetrical migration rates between populations (*M_1_* and *M_2_*). Preliminary runs were performed to set the upper bounds of the prior distribution of each parameter. A stepwise mutation model was used with the following settings for the prior distributions on population parameters: scalars for theta values = 250; maximum migration rates = 100; maximum time of population splitting t = 30. To achieve adequate effective sample size (ESS) numbers and low autocorrelations between parameters [[7](#_ENREF_7)], it was necessary to incorporate 80 chains per step with geometric heating (ha0.99, hb0.75) in each analyses. A total of 1,000,000 steps were retained with the first 1000,000 steps discarded as burn-in. Convergence to the stationary distribution of parameter values and consistency across independent analyses was confirmed with four separate runs with different starting seeds. All parameters had ESS values exceeding 120, indicating convergence was achieved as per the IMa2 guidelines.

**Pathogenicity**

**Pathogenicity trials:** Isolates stored at −80°C were plated onto lima bean agar (LBA) plates (per liter: filtrate of 80g lima beans soaked overnight then autoclaved in 600mL water, 4g malt extract, 4g yeast extract, 4g sucrose, 17g agar, 50mg kanamycin). Plates were incubated for 10 days at 22°C in darkness; isolates were each multiplied onto seven LBA plates and grown for 14 days. Spores were harvested from plates by adding 1.5mL sterile water to each plate and scraping with a sterilised microscope slide. Spores were filtered through doubled cheesecloth then counted using a haemocytometer. Concentrations were adjusted to 5×10^5^ spores mL^−1^.

Barley and barley grass lines were grown for one month in 40-cell Hiko trays, one of each barley cultivar and barley grass lines per tray. The position of each seed line was randomly varied across trays. Barley grass seeds were planted 5 days prior to barley seeds to achieve similar development at inoculation, i.e. the three-leaf stage. Each isolate was inoculated onto two trays of plants using ~100 mL spore suspension with 10 μL Tween-20, spraying until runoff. All inoculations were conducted within a single three-hour period. Following inoculation, the seedlings were placed in the dark with >80 % humidity in a single growth room at 12 ° C for 80 h and then transferred to a glasshouse with night temperatures ranging from 10-13°C and day temperatures from 16-22°C, with pots arranged randomly. Green shade cloth over the glasshouse was used to reduce solar intensity and help maintain temperatures post-infection in late-October growth until the percentage leaf area infected assessments were made 14 d after inoculation using the image analysis software APS Assess [[8](#_ENREF_8)]. A universally susceptible barley cultivar (Sloop) was included to assess inoculation success. All isolates were able to infect Sloop.

**Discussion**

**Sampling bias:** An important consideration, given our goal of estimating host-associated pathogen diversity, was to ensure a comparable sampling effort. This was not entirely possible given unpredicted plant densities and occurrences of *R. commune* on barley grass. Disease incidence of *R. commune* is much lower on barley grass than on barley (Linde observation), which meant host-associated populations could not be sampled in closer proximity than was achieved. It also meant that the distance between sampled infected barley grass plants was often greater than 1 m (1-8 m). Given that dispersal of asexual *R. commune* propagules is likely to be a few meters only [[9](#_ENREF_9)], this constraint on sampling could potentially bias barley grass *R. commune* populations to contain more genotypes (less asexual propagules on a larger scale) than populations from barley. Our results suggest the opposite; therefore, sampling density is unlikely affecting population diversity.

Another sampling bias that could affect genetic diversity estimates, is the geographic area from which most of the barley grass associated pathogen populations were sampled. Five of the seven populations from barley grass occurred in a small geographic area (McLaren Vale; populations approximately 3 to 15 km apart), perhaps inflating genetic relatedness and reducing overall genetic diversity. However, one of these populations (Aldinga) was highly differentiated but had lower differentiation with populations from barley (Table S7). Furthermore, three of the six populations from barley were from a similar size area (Yorke Peninsula) as the barley grass associated populations from McLaren Vale. In addition, no isolation by distance was detected among all populations (R^2^ = 0.0286, *P* = 0.270) or barley grass-associated populations (R^2^ = 0.03433, *P* = 0.270), with only a weak isolation by distance signal in barley-associated populations (R^2^ = 0.2864, *P* = 0.020). Therefore, our sampling strategy is deemed sufficient to enable us to make inferences about the host-associated pathogen diversities.

**Does host genetic diversity and/or pathogen *N* affect pathogen diversity?**

Nevertheless, despite the common finding of disequilibrium, we found fewer MLGs that were distributed equitably and higher linkage disequilibrium in barley grass-associated *R. commune* populations. This contradicts our prediction that *R. commune* should reproduce sexually (have lower linkage disequilibrium) more frequently on barley grass than on barley, following the Red Queen Hypothesis [[1](#_ENREF_1), [2](#_ENREF_2)]. It can be argued that because *R. commune* and *Hordeum* did not coevolve [[10](#_ENREF_10), [11](#_ENREF_11)], the time frame (approx. 150 years in Australia) for evaluating the effect of the Red Queen Hypothesis is too short. However, one of the prerequisites for the Red Queen Hypothesis, that parasites should be adapted to locally common host genotypes, is met in this study. Strong local adaptation of *R. commune* isolates to barley grass genotypes of the same region (McLaren Vale) was found in this (data not shown) and a previous study [[12](#_ENREF_12)]. Again, this study highlights the potentially misleading result that may be achieved when the factors host genetic diversity and population size in maintaining pathogen genetic diversity are studied in isolation.

**References**

1. Clay K, Kover PX: **The red queen hypothesis and plant/pathogen interactions**. *Annu Rev Phytopathol* 1996, **34**:29-50.

2. Ooi K, Yahara T: **Genetic variation of geminiviruses: comparison between sexual and asexual host plant populations**. *Mol Ecol* 1999, **8**:89-97.

3. Linde CC, Zala M, McDonald BA: **Isolation and characterization of microsatellite loci from the barley scald pathogen, *Rhynchosporium secalis***. *Mol Ecol Notes* 2005, **5**:546-548.

4. Linde CC, Zala M, McDonald BA: **Molecular evidence for recent founder populations and human-mediated migration in the barley scald pathogen *Rhynchosporium secalis***. *Mol Phylogenet Evol* 2009, **51**:454-464.

5. Schürch S, Linde CC, Knogge W, Jackson LF, McDonald BA: **Molecular population genetic analysis differentiates two virulence mechanisms of the fungal avirulence gene NIP1**. *Mol Plant-Microbe Interact* 2004, **17**:1114-1125.

6. Linde CC, Zala M, Ceccarelli S, McDonald BA: **Further evidence for sexual reproduction in *Rhynchosporium secalis* based on distribution and frequency of mating-type alleles**. *Fungal Genet Biol* 2003, **40**:115-125.

7. Hey J: **Isolation with migration models for more than two populations**. *Mol Biol Evol* 2010, **27**:905–920.

8. Lamari L: **Assess: Image Analysis Software for Plant Disease Quantification. The American Phytopathological Society, St. Paul, USA.** In*.*; 2002.

9. McDonald BA, Zhan J, Burdon JJ: **Genetic structure of *Rhynchosporium secalis* in Australia**. *Phytopathology* 1999, **89**:639-645.

10. Zaffarano PL, McDonald BA, Linde CC: **Rapid speciation followed host specialization in *Rhynchosporium***. *Evolution* 2008, **62**:1418-1436.

11. Zaffarano PL, McDonald BA, Zala M, Linde CC: **Global hierarchical gene diversity analysis suggests the Fertile Crescent is not the center of origin of the barley scald pathogen *Rhynchosporium secalis***. *Phytopathology* 2006, **96**:941-950.

12. Brown JS: **Pathogenic variation among isolates of *Rhynchosporium secalis* from barley grass growing in south eastern Australia**. *Euphytica* 1990, **50**:81-89.
